# Human aortic allograft: an excellent conduit choice for superior vena cava reconstruction

**DOI:** 10.1186/1749-8090-9-16

**Published:** 2014-01-15

**Authors:** Kristyn Spera, Kenneth A Kesler, Amjadullah Syed, Jack H Boyd

**Affiliations:** 1 Department of Surgery, Division of Cardiothoracic Surgery, Indiana University School of Medicine, 545 Barnhill Dr, EH 215, Indianapolis, IN 46202, USA

**Keywords:** Allograft, Homograft, Venous disease, Revascularization (superior vena cava), Surgery/incisions/exposure/techniques, Mediastinum

## Abstract

Superior vena cava (SVC) reconstruction is occasionally required in the treatment of benign and malignant conditions. We report a patient with symptomatic SVC obstruction secondary to mediastinal fibrosis successfully reconstructed with an aortic allograft.

## Background

Many techniques have been described for SVC reconstruction including the use of spiral saphenous vein, autologous and bovine pericardium, and vascular prostheses, such as externally stented polytetraflouroethylene grafts. Due to wall compliance consistent with vascular tissue, superior handling characteristics, and low thrombogenicity, human cryopreserved arterial allografts have recently been utilized for SVC reconstruction following resection for malignancy [1-3]. We report a patient with symptomatic SVC obstruction secondary to mediastinal fibrosis successfully treated with resection and reconstruction using a thoracic aortic allograft.

## Case presentation

**Figure 1:**
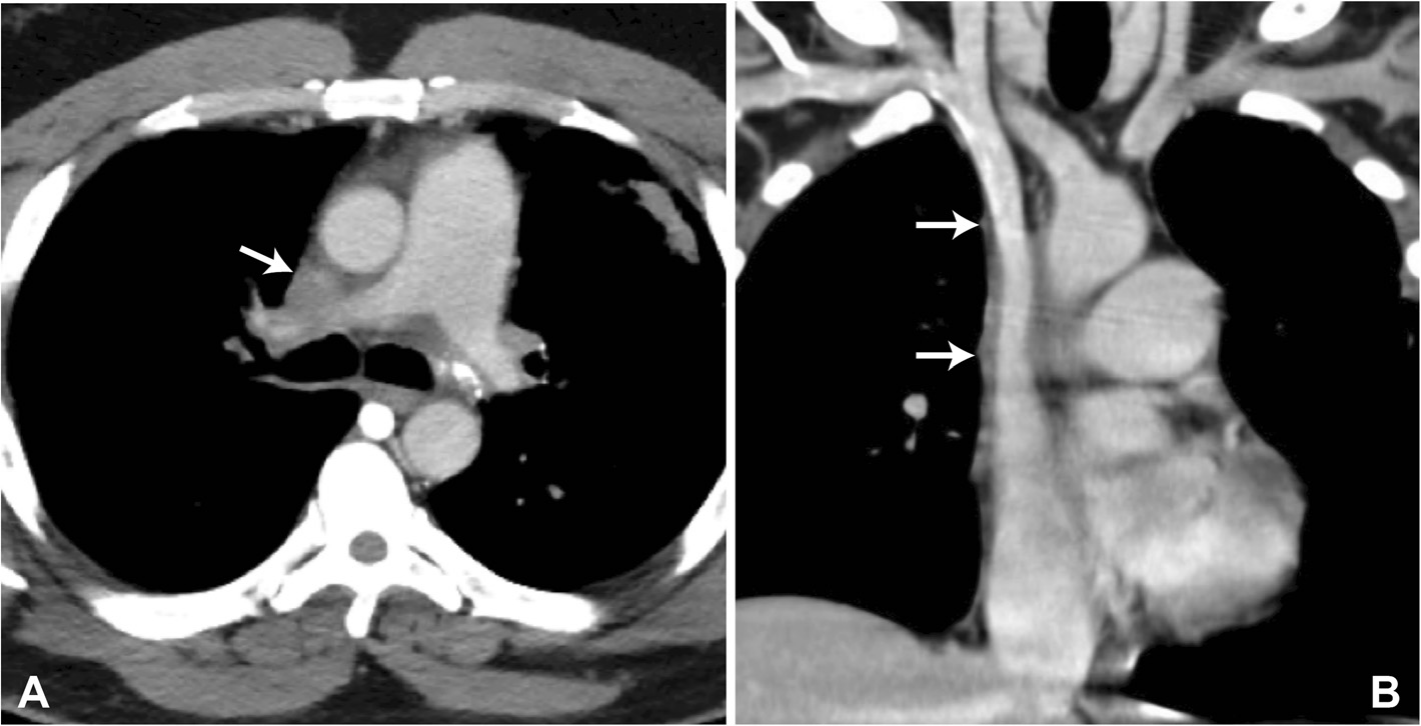
**Pre and postoperative CT images. A**. Preoperative axial computed tomogram with intravenous contrast demonstrating near-total obstruction of the SVC at the level of the right pulmonary artery (arrow). **B**. Postoperative coronal computed tomogram with intravenous contrast demonstrating wide patency of the cryopreserved thoracic aortic allograft-reconstructed SVC (arrows on proximal and distal anastomoses).

**Figure 2:**
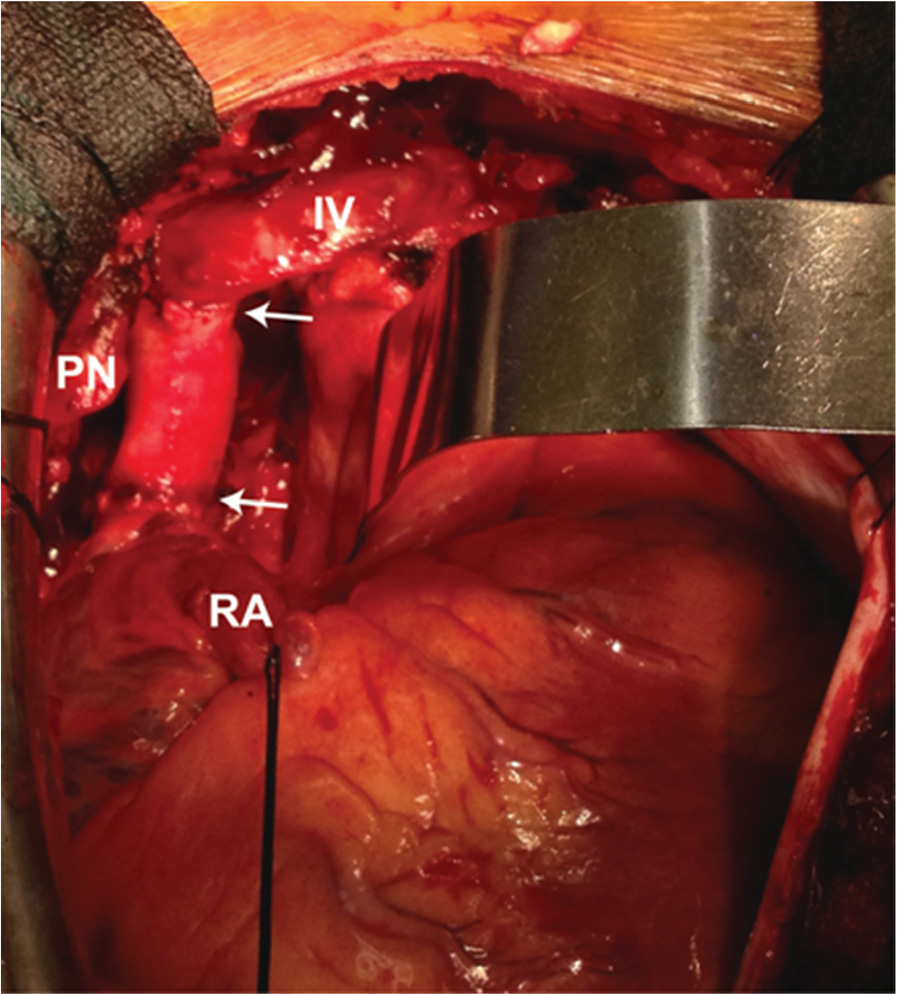
Intraoperative photograph of allograft conduit in the anatomic SVC position (arrows on proximal and distal anastomoses, RA = right atrial appendage, IV = left innominate vein, PN = phrenic nerve and pedicle).

The patient is a 47-year-old African-American male who presented with worsening syncope, brachiocephalic swelling, and dyspnea. Contrast computed tomography (CT) demonstrated near total obliteration of the SVC at the level of the right pulmonary artery with an area of soft tissue calcification consistent with remote granulomatous infection. (Figure 1A) The obstruction was felt unamenable to percutaneous intervention therefore SVC resection and reconstruction planned through a median sternotomy approach. Intraoperatively, large subcutaneous and mediastinal collateral veins were encountered. Circumferential dissection of both innominate veins was ultimately accomplished just proximal to the confluence and the intrapericardial SVC carefully sparing the right phrenic nerve. The azygous vein appeared chronically occluded within the area of fibrosis. Intravenous heparin (100 U/kg) was given and after 3 minutes, both innominate veins and the intrapericardial SVC were clamped one centimeter above the sino-atrial node region. The SVC was divided through the fibrotic tissue and a residual lumen of approximately 1 to 2 mm noted. The SVC and surrounding fibrotic tissue were excised proximally and distally back to normal vessel again with careful preservation of the phrenic nerve, which totaled an approximate 4 to 5 cm segment. Superior vena cava reconstruction was then accomplished using a 16 mm cryopreserved ascending aortic allograft ABO/HLA unmatched (Cryolife Inc, Atlanta, GA). The aortic valve was excised at the top of the commissures, then a proximal end-to-end anastomosis established between the conduit and the proximal SVC, just distal to the innominate confluence, using a running 5–0 polypropylene suture. The allograft was cut to appropriate length, which excised the curvature into the transverse aortic arch. A distal end-to-end anastomosis was performed from the allograft to the SVC approximately 1 cm above the SVC-right atrial junction. Clamps were removed after de-airing to reestablish flow through the homograft. (Figure 2) Subcutaneous heparin (5,000 U) three times daily for DVT prophylaxis and aspirin were begun on the second postoperative day. The patient’s initial postoperative course was uneventful with excellent relief of his symptoms. He was discharged on the seventh postoperative day on aspirin only and remains asymptomatic 12 months following surgery. Pathologic findings of the native SVC and surrounding tissue confirmed an end-stage granulomatous process including fibrosis and calcification most likely due to *Histoplasmosis*. A CT scan with contrast was obtained 6 months after the initial operation confirming patency of the thoracic aortic allograft (Figure 1B).

## Discussion

SVC reconstruction remains a surgical challenge and the optimal method is unknown. All currently utilized methods have limitations, the most significant of which is an inherent risk of thrombosis in a low pressure/flow venous system. We believe there are several advantages of utilizing an allograft for SVC reconstruction. Although controversial, the cryopreservation process of aortic allografts has been shown to potentially diminish endothelial antigenicity with a flow surface more resistant to inflammatory responses and secondary clot formation when used in the arterial system [1-3]. Long-term studies with respect to ABO/HLA matched and unmatched vascular allografts are however needed in this regard. Aortic allografts confer a wall compliance more closely related native vascular tissue. Tissue handling and suturing is superior as compared to other conduits. Finally, aside from thawing, no preparation is necessary. Although the natural curvature of an ascending aorta allograft has been used as a “bypass” conduit from the left innominate vein to the right atrial appendage, we believe that orthotopic SVC reconstruction or direct right innominate vein to SVC reconstruction represents an optimal approach for patients who require resection of the innominate vein confluence. SVC conduits routed orthotopically or right innominate to SVC conduits would likely be more physiologic and less subject to kinking from lung or cardiac motion [4,5].

## Conclusions

We believe this is the first report of an aortic allograft used for SVC reconstruction due to symptomatic obstruction secondary to fibrosing mediastinitis. In light of the benign etiology, the additional expense of an allograft as compared to standard prosthetic vascular grafts can more easily be justified. In this regard, either a descending thoracic or abdominal aorta allograft, if available, may represent a somewhat less expensive option. Based on early experience, further study with allograft tissue for SVC/innominate vein reconstruction is warranted for both benign and malignant conditions.

## Consent

Written informed consent was obtained from the patient for publication of this case report and accompanying images. A copy of the written consent is available for review by the Editor-in-Chief of this journal.

## Abbreviations

SVC: Superior vena cava; CT: Computed tomography; DVT: Deep venous thrombosis; ABO/HLA-ABO: Blood type; HLA: Human leukocyte antigen.

## Competing interests

The authors declare that they have no competing interests.

## Authors’ contribution

KS primarily drafted the manuscript. KK conceived the study and overall manuscript design. AS participated in manuscript writing/revisions. JB participated in manuscript writing/revisions and was responsible for all images. All authors read and approved the final manuscript.
